# Reactive oxygen species drive herpes simplex virus (HSV)-1-induced proinflammatory cytokine production by murine microglia

**DOI:** 10.1186/1742-2094-8-123

**Published:** 2011-09-26

**Authors:** Shuxian Hu, Wen S Sheng, Scott J Schachtele, James R Lokensgard

**Affiliations:** 1 Neuroimmunology Laboratory, Center for Infectious Diseases and Microbiology Translational Research, Department of Medicine, University of Minnesota, Minneapolis, MN, USA

## Abstract

**Background:**

Production of reactive oxygen species (ROS) and proinflammatory cytokines by microglial cells in response to viral brain infection contributes to both pathogen clearance and neuronal damage. In the present study, we examined the effect of herpes simplex virus (HSV)-1-induced, NADPH oxidase-derived ROS in activating mitogen-activated protein kinases (MAPKs) as well as driving cytokine and chemokine expression in primary murine microglia.

**Methods:**

Oxidation of 2', 7'-dichlorodihydrofluorescin diacetate (H_2_DCFDA) was used to measure production of intracellular ROS in microglial cell cultures following viral infection. Virus-induced cytokine and chemokine mRNA and protein levels were assessed using real-time RT-PCR and ELISA, respectively. Virus-induced phosphorylation of microglial p38 and p44/42 (ERK1/2) MAPKs was visualized using Western Blot, and levels of phospho-p38 were quantified using Fast Activated Cell-based ELISA (FACE assay). Diphenyleneiodonium (DPI) and apocynin (APO), inhibitors of NADPH oxidases, were used to investigate the role of virus-induced ROS in MAPK activation and cytokine, as well as chemokine, production.

**Results:**

Levels of intracellular ROS were found to be highly elevated in primary murine microglial cells following infection with HSV and the majority of this virus-induced ROS was blocked following DPI and APO treatment. Correspondingly, inhibition of NADPH oxidase also decreased virus-induced proinflammatory cytokine and chemokine production. In addition, microglial p38 and p44/42 MAPKs were found to be phosphorylated in response to viral infection and this activation was also blocked by inhibitors of NADPH oxidase. Finally, inhibition of either of these ROS-induced signaling pathways suppressed cytokine (TNF-α and IL-1β) production, while chemokine (CCL2 and CXCL10) induction pathways were sensitive to inhibition of p38, but not ERK1/2 MAPK.

**Conclusions:**

Data presented herein demonstrate that HSV infection induces proinflammatory responses in microglia through NADPH oxidase-dependent ROS and the activation of MAPKs.

## Background

Microglia, like other phagocytic cells, generate reactive oxygen species (ROS) as a mechanism to eliminate invading pathogens. Oxygen-containing free radicals such as superoxide (O_2_^-^), the hydroxyl radical (^.^OH), and hydrogen peroxide (H_2_O_2_) are highly reactive. ROS production by microglial cells, while beneficial in clearing invading pathogens from the brain, may also induce irreparable harm through bystander damage to crucial host neural cells. The imbalance between the generation of ROS and the cell's ability to detoxify these same mediators produces a state known as oxidative stress [1]. It is well-established that oxidative stress is an important contributing factor to many pathologic and neurodegenerative processes in the central nervous system (CNS) including HIV-associated neurocognitive disease (HAND), Alzheimer's disease, Parkinson's disease, and Amyotrophic lateral sclerosis [2,3].

It is becoming increasingly clear that ROS are also responsible for mediating many of the secondary mechanisms of tissue damage during and subsequent to viral encephalitis [4]. Herpes simplex virus (HSV)-1 infection of the brain is the leading cause of sporadic viral encephalitis with known etiology [5]. It results in devastating necrotizing acute encephalitis, but may also develop into a chronic inflammatory brain disease with associated neurodegeneration [6,7]. As a result, many of the cytopathic effects observed during viral encephalitis may not simply be due to viral replication, but may also result from host-mediated secondary mechanisms of damage associated with viral clearance including oxidative stress.

In the membrane of phagocytic cells, such as microglia, ROS are generated by the activity of the NADPH oxidase family of enzymes. These NADPH oxidases generate ROS by carrying electrons across membranes from NADPH in the cytosol to an electron acceptor (i.e., oxygen) in the extracellular space or phagosome [8]. This results in toxicity being directed towards the invading pathogen. In addition to their direct toxic effects on invading microbes, ROS are also important second messengers in signal transduction (a phenomenon known as redox signaling). In several models, ROS generated from NADPH oxidase have been demonstrated to affect the redox signaling pathways which stimulate cytokine and chemokine production by microglia [9-11]. NADPH oxidase activity has also been linked to HIV Tat-induced cytokine and chemokine production by microglia, as well as Tat-induced transactivation of the HIV LTR [12,13].

We have previously reported that both human and murine microglial cells are the primary brain cell type responsible for cytokine and chemokine production in response to infection with HSV-1 [14,15]. In the present study, we examined the effect of the inhibition of NADPH oxidase on HSV-induced intracellular signal transduction pathways, as well as downstream cytokine and chemokine production.

## Methods

### Reagents

The following reagents were purchased from the indicated sources: Dulbecco's modified Eagle's medium (DMEM), Hanks' balanced salts (HBSS), penicillin, streptomycin, trypsin, Tween 20, phosphate buffered saline (PBS), poly-L-lysine, Tris, bovine serum albumin (BSA), diphenylene iodonium (DPI), apocynin (APO, Sigma-Aldrich, St. Louis, MO); Iba1 (ionized calcium binding adaptor molecule 1) and Mac-1 antibodies (BD Biosceneces, San Diego, CA); acrylamide/bis-acrylamide gel (Bio-Rad, Hercules, CA); CDP-Star substrate (Applied Biosystems, Foster City, CA); K-Blue substrate (Neogen, Lexington, KY); heat-inactivated fetal bovine serum (FBS, Hyclone, Logan, UT); anti-p38 and -extracellular signal-regulated kinase 1 and 2 (ERK1/2 or p44/42) MAPK antibodies (Cell Signaling, Beverly, MA); recombinant murine interleukin (IL)-1β, tumor necrosis factor (TNF)-α, CCL2 CXCL10, anti-murine TNF-α, IL-1β, CCL2 and CXCL10 antibodies (R&D Systems, Minneapolis, MN); RNase inhibitor, SuperScript™ III reverse transcriptase (Invitrogen, Carlsbad, CA); DNase (Ambion, Austin, TX); random hexmer, and oligo (dT)_12-18 _(Gene Link, Hawthorne, NY); SYBR^® ^Advantage^® ^qPCR premix (ClonTech, Mountain View, CA); dNTPs (GE Healthcare, Piscataway, NJ); 2', 7' -dichlorodihydrofluorescein diacetate (H_2_DCFDA), SB203580 (an inhibitor of p38 MAPK), SB202474 (a negative control for SB203580), U0126 (an inhibitor of MAP kinase kinase [MEK]1/2, upstream of ERK1/2), and U0124 (a negative control for U0126) (EMD Chemicals, Gibbstown, NJ).

### Animals

Female and male BALB/c mice, 8 to 10 weeks old, were purchased from Charles River (Wilmington, MA). These mice were housed in a specific pathogen free room (12-hr light-dark cycle) and had open access to a commercial diet and water. This study was approved by the University of Minnesota Institutional Animal Care, Use, and Research Committee.

### Microglial cell cultures

Microglial cells were prepared as previously described [6,15]. In brief, murine cerebral cortical brain tissues from 1 d-old mice were dissociated after a 30-min trypsinization (0.25%) and plated in 75-cm^2 ^Falcon culture flasks in DMEM containing 10% heat-inactivated FBS and antibiotics. The medium was replenished 1 and 4 days after plating. On day 12 of culture, floating microglial cells were harvested, plated into 96-well (4 × 10^4 ^cells/well) or 12-well (1 × 10^6 ^cells/well) plates, and incubated at 37°C. Purified microglial cell cultures were comprised of a cell population in which > 98% stained positively with Mac-1 and Iba-1 antibodies and < 2% stained positively with antibodies specific to glial fibrillary acidic protein (GFAP), an astrocyte marker.

### Virus

HSV-1 strain 17 syn^+ ^was propagated and titrated using plaque assay on rabbit skin fibroblasts (CCL68; American Type Culture Collection, Manassas, VA).

### Intracellular ROS assay

Production of intracellular ROS was measured using H_2_DCFDA oxidation. Murine microglial cultures seeded (4 × 10^4 ^/well) in 96-well plates or 4-well chamber slides were infected with HSV-1 (MOI = 2.5). At designated time points, cells were washed and incubated with HBSS (with Ca^2+^) containing H_2_DCFDA (20 μM) for 45 min (avoiding light exposure). After incubation, cell culture plates were read using a fluorescence plate reader at Ex_485 _and Em_530 _or viewed and photographed under a fluorescence microscope. Each sample was run in triplicate and sample means were normalized to their respective controls (% of control).

### Real-time PCR

One μg of total RNA extracted from microglia after treatment was treated with DNase and reverse transcribed to cDNA with oligo (dT)_12-18_, random hexmer, dNTPs, RNase inhibitor and SuperScript™ III reverse transcriptase. Mixtures of diluted cDNA, primers and SYBR^® ^Advantage^® ^qPCR premix were subjected to real-time PCR (Stratagene, La Jolla, CA) according to manufacturer's protocol. Primer sequences were sense 5'-TGCTCGAGATGTCATGAAGG-3' and antisense 5'-AATCCAGCAGGTCAGCAAAG-3' for HPRT; sense 5'-GCCTCTTCTCATTCCTGCTTGT-3', antisense 5'- CACTTGGTGGTTTGCTACGAC-3' for TNF-α; sense 5'-AGACTTCCATCCAGTTGCCTTC-3' and antisense 5'-CATTTCCACGATTTCCCAGAG-3' for IL-6; sense 5'- AGGCTGGAGAGCTACAAGAGGA-3' and antisense 5'-GACCTTAGGGCAGATGCAGTTT-3' for CCL2; sense 5'-GTCATTTTCTGCCTCATCCTGCT-3' and antisense 5'-GGATTCAGACATCTCTGCTCATCA-3' for CXCL10. The relative mRNA expression levels were quantified using the 2^(-ΔΔCT) ^method [16] and were normalized to the housekeeping gene hypoxanthine phosphoribosyl transferase (HPRT; NM_013556).

### ELISA

In brief, 96-well ELISA plates pre-coated with goat or rabbit anti-mouse cytokine/chemokine antibody (2 μg/ml) overnight at 4°C were blocked with 1% BSA in PBS for 1 h at 37°C. After washing with PBS containing Tween 20 (0.05%), culture supernatants and a series of dilution of cytokines/chemokines (as standards) were added to wells for 2 h at 37°C. Anti-mouse cytokine/chemokine detection antibodies were added for 90 min followed by addition of anti-IgG horseradish peroxidase conjugate (1:10, 000) for 45 min. The chromogen substrate K-Blue was added at room temperature for color development which was terminated with 1 M H_2_SO_4_. The plate was read at 450 nm and cytokine/chemokine concentrations were extrapolated from the standard concentration curve.

### Western Blot

Cell lysates collected after treatment were electrophoresed in 12% acrylamide/bis-acrylamide, electrotransferred onto nitrocellulose membrane and probed with antibodies for phospho-p38 (Thr180/Tyr182) and phospho-p44/42 (Thr202/Tyr204) MAP kinase followed by alkaline phosphatase-conjugated secondary antibodies with chemiluminescence detection using Kodak Image Station (Carestream Health (formerly Kodak), New Heaven, CT). Levels of phosphor-p38 (T180/Y182) and total p38 MAPK were measured using a Fast Activated Cell-based ELISA (FACE™), in-cell Western analysis according to the manufacturer's instructions (Active Motif, Carlsbad, CA).

### MAPK inhibition

Microglial cell cultures were pretreated with SB203580, SB202474, U0126 or U0124 for 1 h prior to viral infection followed by collection of cell culture supernatants for ELISA.

### Statistical analysis

Data are expressed as mean ± SD or SEM as indicated. For comparison of means of multiple groups analysis of variance (ANOVA) was used followed by Scheffe's test.

## Results

### Viral infection induces intracellular ROS generation by murine microglia

**Figure 1:**
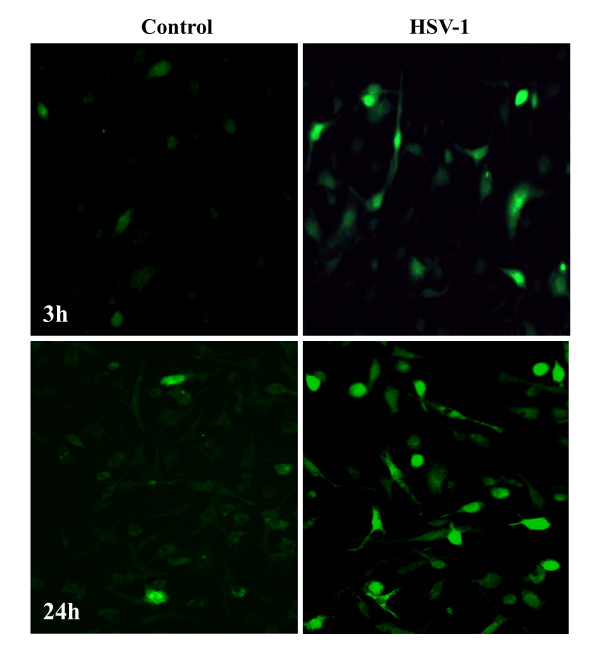
**Intracellular ROS generation in response to HSV-1 infection of primary microglia**. Purified murine microglial cell cultures were either left uninfected (Control) or infected with HSV-1 (MOI = 2.5) for 3 or 24 h prior to loading with H_2_DCFDA (20 μM, 45 min) for visualization using fluorescence microscopy. Data shown are representative of five individual experiments using microglial cells obtained from different animals.

To determine the role of redox responses in virus-induced cytokine and chemokine production, we first examined ROS production by HSV-stimulated microglia. Purified murine microglial cell cultures were infected with HSV at an MOI = 2.5. Virus-induced changes in intracellular ROS levels were assessed through loading the cells with the ROS fluorescence indicator H_2_DCFDA and examination by fluorescence microscopy. In these studies, viral infection was found to induce rapid generation of microglial cell-produced ROS, as early as 3 h, with robust levels evident in most cells by 24 h p.i. (Figure 1). The concentration of H_2_DCFDA used in these experiments (i.e., 20 μM) did not induce microglial cell toxicity as determined by MTT assay and trypan blue staining. In addition, MTT assay was used to check cell viability following viral infection and showed approximately 15% and 40% decreases at 24 and 48 h p.i., respectively.

### Inhibition of NADPH oxidase blunts virus-induced ROS production

**Figure 2:**
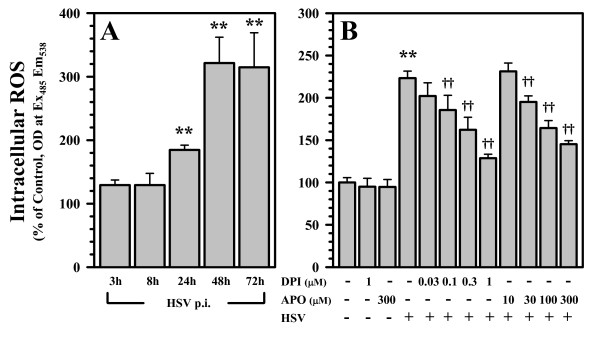
**Inhibition of NADPH oxidase blunts virus-induced ROS production**. Microglia were A) infected with HSV-1 for the designated time or B) left untreated or pretreated with the NADPH oxidase inhibitors DPI (0.03 - 1 μM) or APO (10 - 300 μM) at the indicated concentrations for 1 h prior to viral infection for 36 h, followed by addition of H_2_DCFDA (20 μM) for 45 min and quantification using a fluorescent microplate reader. Data are presented as mean ± SEM from 6-8 separate experiments. **p < 0.01 vs. control; ^†^p < 0.05 and ^††^p < 0.01 vs. HSV alone.

We then went on to examine virus-induced ROS production over a time-course of infection. In these experiments, microglial cells were stimulated with HSV for the designated time, followed by quantification of H_2_DCFDA oxidation using a fluorescence plate reader. Using this microplate assay, ROS levels in microglial cell cultures were found to be elevated by 24 h p.i., and reached maximal levels by 48 h (Figure 2A). We went on to investigate the effect of inhibition of NADPH oxidase on the production of this HSV-induced ROS. In these experiments, microglia were pretreated with the NADPH oxidase inhibitors DPI or APO for 1 h prior to viral stimulation. HSV-induced ROS production was significantly decreased by DPI in a concentration-dependent manner and by APO at 300 μM following the inhibition of NADPH oxidase (Figure 2B). The concentrations of DPI or APO used did not themselves induce microglial cell toxicity as determined by MTT assay and trypan blue staining.

### ROS drive cytokine and chemokine expression in virus-infected microglia

**Figure 3:**
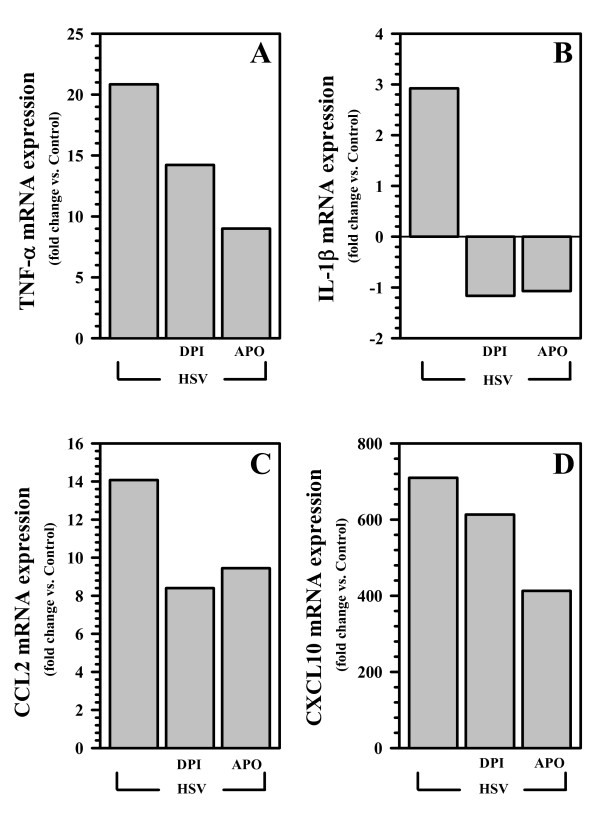
**ROS drive cytokine and chemokine mRNA expression in virus-infected microglia**. Microglial cell cultures were pre-treated with the NADPH oxidase inhibitors DPI or APO for 1 h prior to a 5 h exposure to HSV. Following viral infection, RNA was extracted and cDNA synthesized to assess mRNA expression through quantitative real-time PCR for A) TNF-α; B) IL-1β; C) CCL2; and D) CXCL10. mRNA levels were normalized to the housekeeping gene HPRT and are presented as fold induction over uninfected controls. Data shown are representative of three individual experiments using microglial cells obtained from different animals.

**Figure 4:**
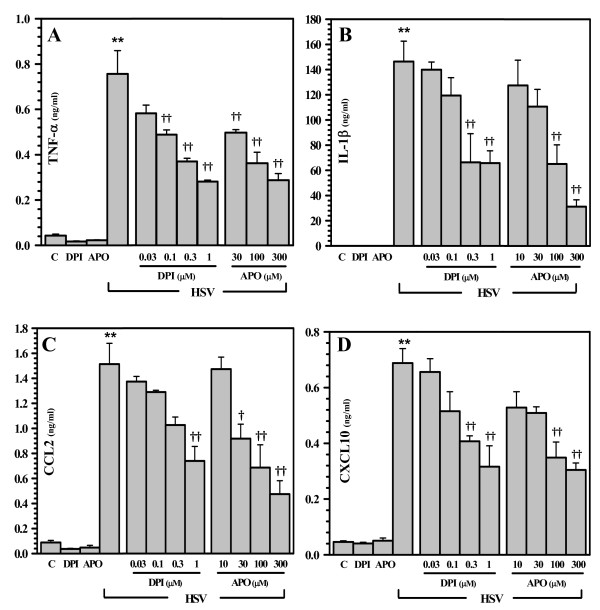
**ROS contribute to cytokine and chemokine production by microglia in response to viral infection**. Supernatants were collected from murine microglial cell cultures pretreated with DPI or APO at the indicated concentrations for 1 h prior to viral exposure for 36 h (or 16 h for TNF-α) and cytokine and chemokine levels were assessed using ELISA for A) TNF-α; B)IL-1β; C) CCL2; and D) CXCL10. Data are presented as mean ± SD of 3 replicates from 3 separate experiments. **p < 0.01 vs. uninfected control; ^†^p < 0.05 and ^††^p < 0.01 vs. HSV alone.

We have previously reported that HSV stimulation of both human and murine microglial cells initiates robust cytokine and chemokine production [14,15]. Data presented here demonstrate that ROS production by microglial cells occurs within 3 h following HSV infection. We've previously reported that cytokine and chemokine mRNA is first detectable using RT-PCR by 5 h p.i. and protein is first detectable by ELISA within 8 h p.i. [15]. The involvement of ROS in driving virus-induced expression of these immune mediators was investigated by pretreatment of microglial cells with DPI (0.03 - 1 μM) and APO (10 - 300 μM) and then using real-time RT-PCR to assess gene expression for select cytokines and chemokines. Treatment with either inhibitor of NADPH oxidase (i.e., DPI or APO) was found to inhibit TNF-α, interleukin (IL)-1β, CCL2, and CXCL10 mRNA expression at 5 h p.i. (Figure 3A-D). We went on to assess the involvement of NADPH oxidase and ROS in cytokine and chemokine production using ELISA to measure protein levels in cell culture supernatants. Corresponding to our findings at the mRNA level, both inhibitors of NADPH oxidase blunted cytokine (TNF-α and IL-1β) and chemokine (CCL2 and CXCL10) protein production in virus-infected microglial cultures (Figure 4A-D).

### Viral infection activates p38 and p44/42 (ERK1/2) MAPKs in primary microglia cells

**Figure 5:**
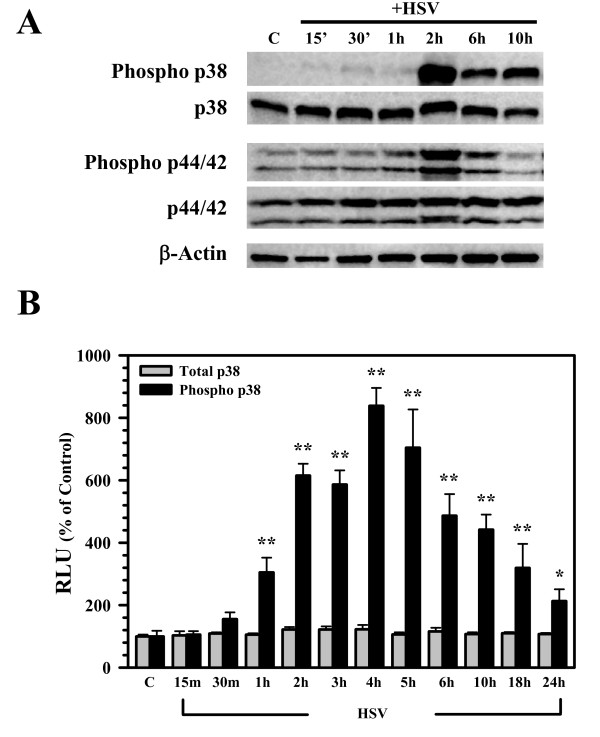
**Activation of p38 and p44/42 (ERK1/2) MAPKs in response to viral infection of primary microglia**. A) Control uninfected (C) or virus-infected (+HSV) microglial cell culture lysates were collected at the indicated time points to assess MAPK activation using Western Blot. B) The kinetics of p38 MAPK activation were quantified in microglial cell cultures infected with HSV-1 using a FACE™ p38 Chemi, in-cell Western assay (Active Motif, Carlsbad, CA). Data presented are representative of mean ± SD with 3 replicates from 2 separate experiments. *p < 0.05 and** p < 0.01 vs. uninfected control.

Activation of MAPKs plays an essential role in the cytokine response of microglial cells to inflammatory stimuli. p38 MAPK has recently been shown to be critical for the neurotoxic phenotype of monocytic cells following exposure to HIV gp120 [17]. For this reason, we examined whether HSV infection activated p38 and p44/42 MAPKs in our primary murine microglia. Using Western Blot, viral infection of primary microglial cells was found to stimulate phosphorylation of both kinases by 2 h p.i. (Figure 5A). These results were confirmed using a more quantifiable FACE in-cell Western assay over a 24 h time-course of infection. Using this assay, significant phosphorylation of p38 MAPK in response to viral infection was detected as early as 1 h p.i., with prolonged activation evident at 24 h p.i. (Figure 5B).

### Redox signaling drives the p38 MAPK activation

**Figure 6:**
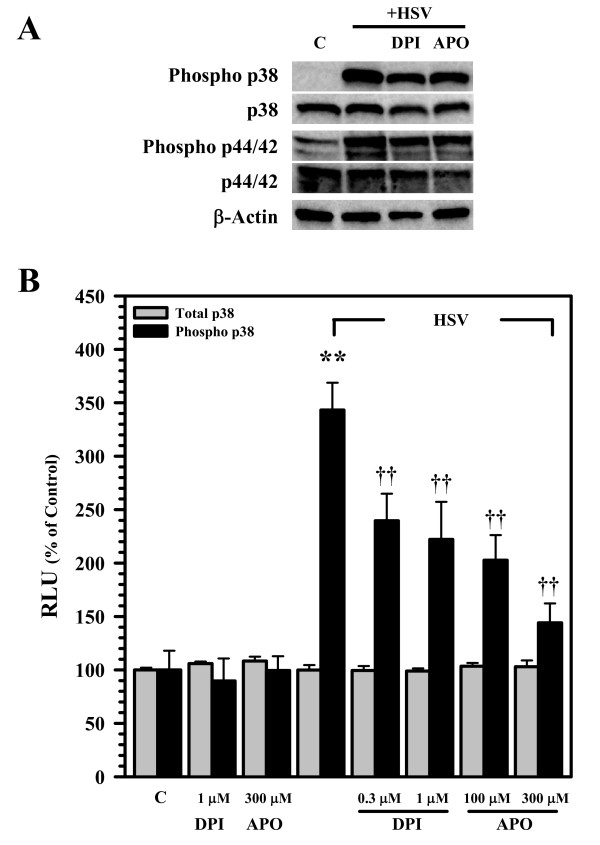
**Redox signaling drives p38 MAPK activation**. A) Cell lysates from uninfected control (C) or virus-infected (+HSV) microglial cells, pretreated with either DPI (1 μM) or APO (300 μM), were collected at 2 h post-infection and MAPK activation was assessed using Western Blot. B) The effect of NADPH oxidase inhibitors (1 h pretreatment) on virus-induced activation of p38 MAPK was quantified 2 h post-infection using a FACE assay. Data are presented as mean ± SD of triplicates and are representative of 2 separate experiments. **p < 0.01 vs. uninfected control; ^††^p < 0.01 vs HSV alone.

We went on to examine the effect of NADPH oxidase and ROS production on MAPK activation in response to viral infection. In these studies, treatment of microglial cells with either DPI or APO prior to viral infection blunted HSV-induced MAPK phosphorylation as detected using Western Blot at 2 h p.i. (Figure 6A). Additionally, FACE assay analysis at 2 h p.i. confirmed that either DPI or APO treatment significantly reduced phosphorylation of p38 MAPK (Figure 6B).

### MAPK inhibition blocks cytokine and chemokine production

**Figure 7:**
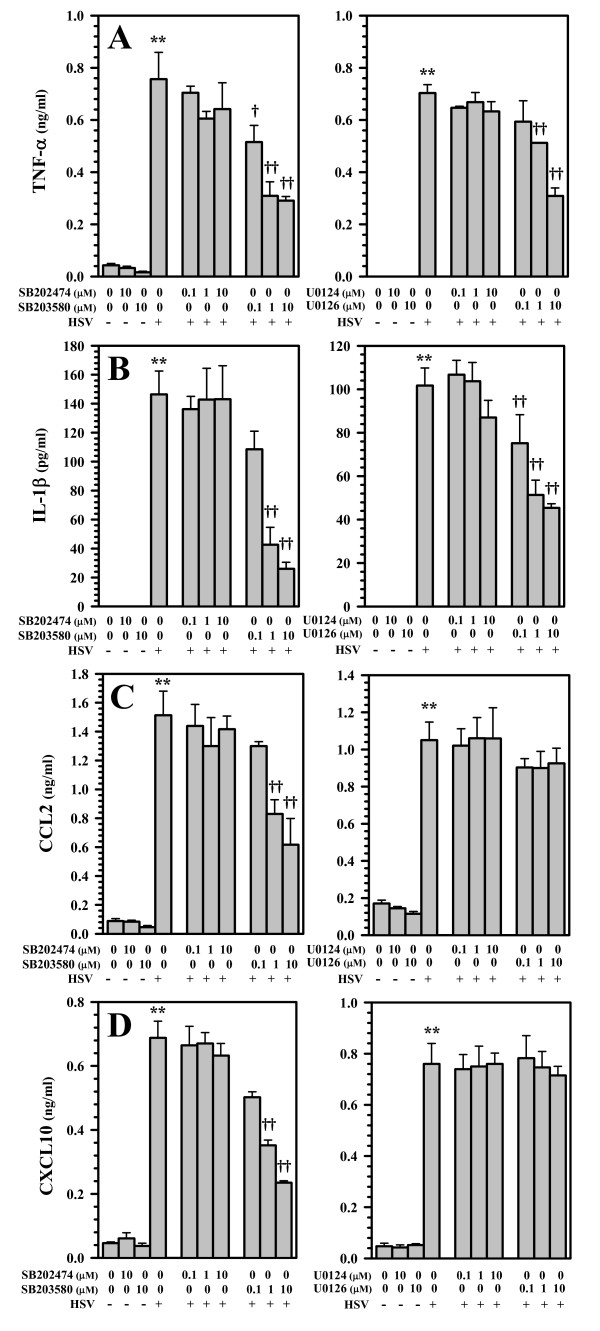
**Involvement of p38 and p44/42 (ERK1/2) in cytokine and chemokine production by virus-infected primary murine microglia**. Microglial cell cultures pretreated with inhibitors of p38 (SB203580 or its negative control SB202474) or ERK1/2 (U1026 or its negative control U0124) MAPKs for 30 min prior to viral infection. At 16 h p.i., supernatants were collected and assessed for A) TNF-α or 36 h for B) IL-1β, C) CCL2, and D) CXCL10 production using ELISA. Data presented are representative of mean ± SD with 3 replicates of 2 separate experiments. **p < 0.01 vs. uninfected control; ^†^p < 0.05 and ^†† ^p < 0.01 vs. HSV alone.

In the last set of experiments, we examined the involvement of these two ROS-driven MAPK signaling pathways in cytokine and chemokine production by microglia in response to viral infection. In these studies, inhibition of the p38 MAPK signaling pathway using SB203580 (0.1 to 10 μM) was found to suppress both cytokine (TNF-α and IL-1β) and chemokine (CCL2 and CXCL10) production (Figure 7). In contrast, inhibition of p44/42 MAPK signaling using U0126 (0.1 to 10 μM) inhibited cytokine (Figure 7A, B), but not chemokine production (Figure 7C, D). Additional assays tested whether MAPK inhibition affected HSV-induced ROS production itself. Data generated from these studies showed that the ERK1/2 (p44/p42) inhibitor U0126 partially suppressed ROS production by 11.1%, 18.1%, and 20.9%, at 0.1, 1.0, and 10 μM, respectively. Correspondingly, the p38 MAPK inhibitor SB203580 also partially suppressed ROS production by 16.3%, 21.1%, and 42.4%, at 0.1, 1.0, and 10 μM, respectively.

## Discussion

We have recently reported that HSV-induced ROS production by microglial cells is responsible for lipid peroxidation, oxidative damage, and toxicity to neurons in culture, and that viral recognition is mediated, at least in part, through Toll-like receptor (TLR)-2 [18]. In several other systems, engagement of TLRs has been demonstrated to induce NADPH oxidase activation, with corresponding ROS generation, which subsequently activates NF-κB to induce proinflammatory cytokine production [19-21]. Following up on our previous work, the present study examined the effect of HSV-1-induced, NADPH oxidase-derived ROS in activating mitogen-activated protein kinases (MAPKs) and driving cytokine, as well as chemokine, expression in primary murine microglia. Data obtained during these studies clearly demonstrate that intracellular ROS are generated following viral infection of murine microglia and are associated with a marked increase in the expression of NADPH oxidase mRNA. Viral infection was found to induce microglial cell-produced ROS as early as 3 h in individual cells, however, additional time was required to reach statistical significance when the entire culture was assessed.

ROS are important second messengers in redox signaling. Viral brain infection initiates robust inflammatory responses pivoting on the production of cytokines and chemokines by microglial cells [15]. We have previously reported that microglial cells undergo an abortive, non-productive infection with HSV-1 in which immediate early gene (e.g., ICP4) expression occurs, but late gene expression (e.g., such as glycoprotein D, gD) and viral replication are blocked [15]. These cells respond to HSV infection by inducing a burst of cytokine and chemokine production, followed by apoptotic death. It has previously been reported that microglial ROS, produced largely through the action of NADPH oxidases, precedes cytokine and chemokine production in response to HIV Tat or *M. tuberculosis *30-kDa Ag [12,22]. In the present study, inhibition of NADPH oxidase with either DPI or APO was also found to decrease subsequent HSV-induced cytokine and chemokine production. These data demonstrate that NADPH-derived ROS drive cytokine and chemokine expression by microglia in response to viral infection.

Phosphorylation of p38 and p44/p42 ERK1/2 MAPK is commonly associated with TLR signaling and has been implicated in TLR-associated ROS production [11,19,23,24]. Because these MAPKs play an important role in regulating the expression of immune mediators following stimulation with viruses, viral proteins, and other inflammatory factors [9,14,17,25-27], we next investigated the role of p38 and p44/p42 ERK1/2 activation in HSV-infected microglia. In these studies, we first found that viral infection induced the phosphorylation of both MAPKs. We then went on to perform experiments using the inhibitors DPI and APO to determine whether NADPH oxidase-derived ROS drive viral activation of p38 and p44/p42 ERK1/2 MAPKs. In these studies, treatment of microglial cells with the NADPH oxidase inhibitors was found to blunt HSV-induced MAPK phosphorylation by Western Blot (p38 and p44/p42 ERK1/2) and FACE (p38) assay.

In our last set of experiments we investigated the effect of blocking specific MAPK pathways on HSV-induced cytokine and chemokine production. Using human microglia, we have previously reported that while an inhibitor of p38 MAPK (SB202190) blocked both HSV-induced cytokine and chemokine production, treatment with the ERK1/2 inhibitor (U0126) inhibited the induction of cytokines (i.e., TNF-α, IL-1β), but not chemokines (i.e., CCL5 and CXCL10), [14]. In the present study, very similar differential cytokine and chemokine results are found using HSV-infected murine microglia. HSV-induced TNF-α and IL-1β production was found to be susceptible to inhibition by both the p38 MAPK inhibitor SB203580 and the p44/p42 ERK1/2 inhibitor U0126, while virus-induced CXCL10 and CCL2 was suppressed by SB203580, but the p44/p42 ERK1/2 inhibitor had no inhibitory effect at any concentration tested. Taken together, it is likely that insufficient activation of these MAPK pathways following the inhibition of NADPH oxidase, and decreased ROS generation, is responsible for the attenuated cytokine production.

A number of studies have shown that beneficial neuroimmune responses, for example those needed to purge infectious virus from the brain, can develop into chronic pathological inflammation with progressive neurodegeneration [28]. Restoration of redox balance may be an important determinant in returning activated microglia back to a resting state following viral infection and neuroinflammation. The findings presented herein support the idea that ROS-driven microglial cell activation, and its associated neurotoxicity, may be a target for therapeutic modulation through the stimulation of opposing anti-oxidative responses.

## Competing interests

The authors declare that they have no competing interests.

## Authors' contributions

SH co-conceived of the study, and designed and performed experiments. WS performed experiments and analyzed data. SJS participated in study design. JRL co-conceived of the study, participated in its design, and wrote the manuscript. All authors have read and approved the final manuscript.
